# Approaches and experiences implementing remote, electronic consent at the Leeds Clinical Trials Research Unit

**DOI:** 10.1186/s13063-024-08149-y

**Published:** 2024-05-08

**Authors:** William J Cragg, Chris Taylor, Lauren Moreau, Howard Collier, Rachael Gilberts, Niamh McKigney, Joanna Dennett, Sandra Graca, Ian Wheeler, Liam Bishop, Adam Barrett, Suzanne Hartley, John P Greenwood, Peter P Swoboda, Amanda J Farrin

**Affiliations:** 1 Clinical Trials Research Unit, Leeds Institute of Clinical Trials Research, University of Leeds, Leeds, UK; 2 Leeds Institute of Cardiovascular and Metabolic Medicine, University of Leeds, Leeds, UK

**Keywords:** Electronic consent, Remote consent, eConsent

## Abstract

**Background:**

Use of electronic methods to support informed consent (‘eConsent’) is increasingly popular in clinical research. This commentary reports the approach taken to implement electronic consent methods and subsequent experiences from a range of studies at the Leeds Clinical Trials Research Unit (CTRU), a large clinical trials unit in the UK.

**Main text:**

We implemented a remote eConsent process using the REDCap platform. The process can be used in trials of investigational medicinal products and other intervention types or research designs. Our standard eConsent system focuses on documenting informed consent, with other aspects of consent (e.g. providing information to potential participants and a recruiter discussing the study with each potential participant) occurring outside the system, though trial teams can use electronic methods for these activities where they have ethical approval. Our overall process includes a verbal consent step prior to confidential information being entered onto REDCap and an identity verification step in line with regulator guidance. We considered the regulatory requirements around the system’s generation of source documents, how to ensure data protection standards were upheld and how to monitor informed consent within the system.

We present four eConsent case studies from the CTRU: two randomised clinical trials and two other health research studies. These illustrate the ways eConsent can be implemented, and lessons learned, including about differences in uptake.

**Conclusions:**

We successfully implemented a remote eConsent process at the CTRU across multiple studies. Our case studies highlight benefits of study participants being able to give consent without having to be present at the study site. This may better align with patient preferences and trial site needs and therefore improve recruitment and resilience against external shocks (such as pandemics). Variation in uptake of eConsent may be influenced more by site-level factors than patient preferences, which may not align well with the aspiration towards patient-centred research. Our current process has some limitations, including the provision of all consent-related text in more than one language, and scalability of implementing more than one consent form version at a time. We consider how enhancements in CTRU processes, or external developments, might affect our approach.

## Background

Use of electronic, rather than paper-based, methods to support the different elements of informed consent (‘eConsent’) is an increasingly popular approach in clinical trials, particularly as a result of the COVID-19 pandemic [1]. Suggested benefits include informing patients more efficiently and effectively prior to their decision about taking part, increased inclusivity and improved trial recruitment [2–4]. eConsent is also a key facilitator for conducting ‘decentralised’ clinical trials [5]. However, the potential benefits to recruitment and inclusivity likely only apply where eConsent is used in addition to paper-based consent rather than as a replacement, as digital methods are excluding to some. Others have also raised concerns about data protection around eConsent [1].

The Clinical Trials Research Unit (CTRU) at the University of Leeds is a large clinical trials unit in the UK and a member of the UK Clinical Research Collaboration Registered Clinical Trials Unit Network. The CTRU runs a range of trials and other studies, often with innovative designs, including clinical trials of investigational medicinal products (CTIMPs), complex intervention trials and surgical trials. The populations of people who might take part in CTRU studies are therefore diverse.

From the CTRU’s inception in 1992 until 2021, tens of thousands of patients gave consent to take part in CTRU trials, all using paper-based consent systems. In that year, partly in response to the COVID-19 pandemic, we began using eConsent methods in some of our studies. This commentary reports our approach and our experiences so far.

## The CTRU eConsent approach

### Choice of platform and system scope

We implemented a remote eConsent process alongside our existing paper-based processes. We used the REDCap (Research Electronic Data Capture) software platform, similarly to many other academic clinical trials units in the UK [1]. We chose REDCap [6] as it has an existing eConsent module and was already in use at the CTRU for other aspects of trial delivery. We considered it was likely to be relatively easy for patients to use and could be accessed on a variety of devices and using accessibility tools such as screen readers. REDCap’s availability at no cost [7] was particularly appealing whilst we explored the use of REDCap for eConsent in our studies, before committing to the cost of other systems.

A multi-disciplinary working group, representing the different research divisions within the CTRU, was set up to agree the details of the eConsent process. Using available guidance, including from the UK Medicines and Healthcare products Regulatory Agency (MHRA) and Health Research Authority (HRA) [8], we implemented an eConsent system in REDCap capable of supporting consent in both CTIMPs and non-CTIMPs. Our eConsent system can also support separate registration and randomisation steps within a study and ‘reconsent’ where participants are asked to formally update their consent to new trial information at a subsequent point within the trial.

### Process design

**Fig. 1:**
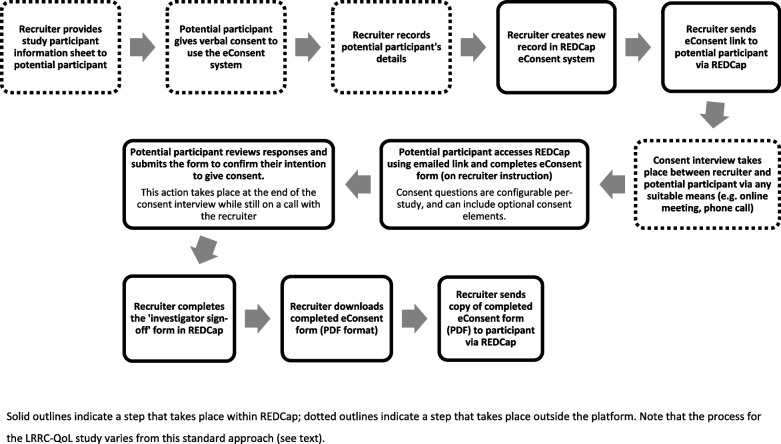
Standard process flow for eConsent at the Leeds Clinical Trials Research Unit. Solid outlines indicate a step that takes place within REDCap; dotted outlines indicate a step that takes place outside the platform. Note that the process for the LRRC-QoL study varies from this standard approach (see text)

**Fig. 2:**
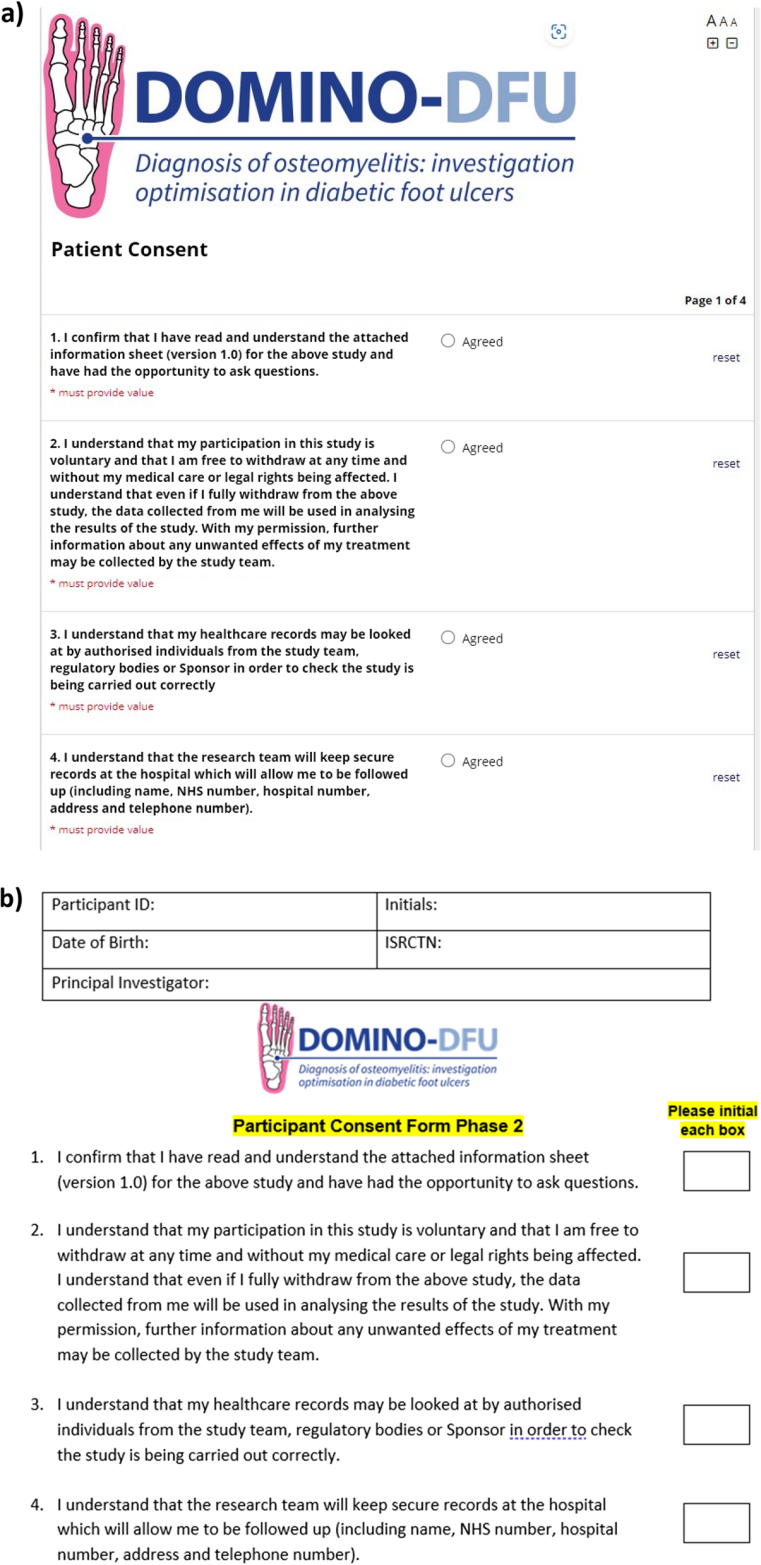
Illustrative screen shots showing **a** the eConsent system and **b** the paper-based equivalent from the DOMINO-DFU study

Figure 1 shows an overview of our overall eConsent process, including the steps that take place outside the REDCap system. Fig. 2 shows illustrative screenshots from the eConsent system and from the Microsoft Word document that is used to create the paper version.

Throughout this article, ‘system’ means our use of the eConsent module in REDCap, and ‘process’ means the whole eConsent process, including steps outside of REDCap. In our standard process, the parts performed by a ‘recruiter’ are done by authorised research staff at recruiting trial sites (e.g. UK National Health Service Organisations). CTRU staff set up the system and oversee its operation but are not typically directly involved in recruitment.

‘Remote’ here means the process is designed for patients and research staff to be in different physical locations. Our focus on remote eConsent was motivated by the need to facilitate trial continuation, despite limitations on in-person interactions during the COVID-19 pandemic. In addition, our use of REDCap eConsent relies on patients accessing personal email accounts or smartphones to access the link to the system. This created logistical challenges we were not initially in a position to resolve for the purposes of implementing an efficient in-person eConsent process.

eConsent has been noted to potentially support multiple aspects of informed consent, including information provision, comprehension assessment and obtaining a valid signature [9]. REDCap does not have functionality for online meetings, so elements of the process that rely on discussion are not done within our eConsent system. Similarly, our standard use of the platform does not include electronic means of conveying information. As REDCap’s eConsent module cannot easily support provision of site-specific information (e.g. information with local contact details, or where different sites are using different patient information sheet versions simultaneously during the trial), we focused only on documenting informed consent. In addition, although it is possible to add or link to study information directly in REDCap [10], we agreed that potential participants needed to receive information about the trial prior to accessing the eConsent system. This was partly to ensure they had time to absorb the information in their own time and partly because they would not yet have agreed for their details to be added to the system. We have diverged from this standard approach in one lower-risk study, however (see “The LRRC-QoL study” section below). Trial teams may nonetheless communicate trial information via electronic means outside of the eConsent system, for example via videos or other media, where they have approval for these.

Prior to beginning the REDCap aspects of the eConsent process, research staff introduce the trial to the patient as they usually would (e.g. during routine clinical contact). If the patient wants to consider giving consent to participate, they are offered remote eConsent or paper-based consent (which can also be done remotely in some cases). We ensure there is a paper process in place as a backup in case the eConsent system is not accessible or not functioning as planned. If participants wish to withdraw their consent after having given it via consent via the eConsent system, this is done by notifying the research staff directly as advised in the patient information sheet (i.e. there is no way to record withdrawal of consent via eConsent).

### Regulatory issues, data protection and confidentiality

In our paper-based consent process, confidential patient information is not disclosed to the CTRU until a patient has consented to take part in a trial. In the eConsent process, confidential information (namely implied information about individuals’ health, often rendered identifiable by individuals’ email addresses) is effectively disclosed to CTRU at the point when a patient’s data is added to REDCap. For this reason, we included a simple initial step for patients to verbally consent to this confidential information being disclosed to CTRU. As the system processes personal data, details about the processing required to be disclosed under the UK General Data Protection Regulation are also provided at this stage in the patient information sheet.

In line with the MHRA/HRA guidance, we incorporated an identity verification step into the process. Our approach to this is proportionate and tailored to the specific patient pathway of each trial [8]. For example, if the patient is likely to already be known to the recruiters, then this can be visually confirmed on a video call and/or by checking basic identifiers with the patient at the start of the consent discussion, such as their name and date of birth. It can also be done in-person at the first trial visit, provided this is before the intervention being administered [8]. Where teams are relying on information to verify identity, then this information needs to be from a different source (i.e. not the same information that the patient provided at a previous interaction). In any case, sites are instructed to document the identity verification step when the remote eConsent process is used.

Records of each participant’s eConsent are source documents, from a regulatory point of view. Our system ensures compliance with ALCOA+ principles [11] regarding source data. For example, all data is attributable to a logged-in recruiter or to a participant’s email address, and an audit trail retains the original data and shows details of any changes made (in the rare cases where changes could be justified). Use of an electronic system inherently supports ALCOA+ principles, for example ensuring data is legible and complete. Completed forms are locked and made read-only and cannot be changed without invalidating the electronic signature.

We implemented data protection ‘by design and default’ [12] in designing the eConsent system. All collected data is encrypted during transit and rest using Advanced Encryption Standard 256. Data, including in backup, is stored on UK-based servers. Data is backed-up nightly. eConsent data is stored separately to any other trial data collected about each individual, and access controls are applied so that only those who need access to particular data, have access. Completed consent forms sent to participants are not encrypted as we considered this to be a likely barrier to the forms being accessible; however, the email address used is already verified through the potential participant’s involvement in the eConsent process, so the chance of it going to an incorrect recipient is minimised. Data is not retained for patients who do not ultimately consent, or are not recruited to the study, beyond the time when this decision is confirmed.

CTRU has well-established central monitoring processes for confirming informed consent is in place in trials using paper-based consent methods. We do this by securely collecting copies of consent forms (with participants’ prior consent for this disclosure) in order to perform checks around the time of recruitment. We updated this monitoring to allow for eConsent. This revision resulted in a reduced series of checks, including that each eConsent record is complete, checking reasons for any date discrepancies (and collecting a reason for discrepancies upfront, unlike on paper forms), checking and managing any incorrect email addresses and noting where records may need to be deleted (where there is confirmation that the patient will not consent, or where this needs to be checked with the trial site staff).

### Case studies

So far, two randomised trials, one multiphase clinical cohort study and one mixed methods study have implemented remote eConsent (see Table 1 for summary details of these projects). We summarise experiences in each of these studies below, with exploratory data presented where available. Although a particular need has recently been highlighted for more evidence around eConsent in clinical trials [1], we have chosen to report all these case studies to help grow the evidence base around eConsent.

**Table 1:** Summary information about studies included as case studies in this commentary

**Study name**	**Full title and ISRCTN details**	**Study design**	**Population**	**Setting**	**Recruitment start and end dates**	**Other study details**	**Other relevant features**	**Use of eConsent**
MODULATE	Management of diarrhoea in ulcerative colitis: multi-arm multi-stage trial of low FODMAP diet, amitriptyline, ondansetron, or loperamide—ISRCTN16086699	Randomised controlled multi-arm, multi-stage trial	Patients with ulcerative colitis and diarrhoea	Planned to open in around 26 secondary care sites in the UK	Start and end: December 2021 (eConsent implemented after trial opened to recruitment, but prior to recruitment of first participant)	Trial comparing dietary and drug interventions to a control of standard first-line dietary advice. Clinical Trial of an Investigational Medicinal Product (CTIMP) Primary outcome: proportion of participants achieving improvement in discomfort from diarrhoea on Gastrointestinal Symptom Rating Scale-Irritable Bowel Syndrome questionnaire at 8 weeks	Trial reconfigured after initial pre-COVID approvals to incorporate a remote trial delivery pathway Began recruitment but closed early	1 participant recruited to the study and they used eConsent
CE-MARC 3	A pragmatic approach to the investigation of stable chest pain: a UK, multi-centre, randomised trial to improve patient experience, outcomes and NHS cost efficiency—ISRCTN88179970	Randomised controlled trial	Patients with new onset chest pain requiring further investigation	Cardiology departments at 8–15 secondary care sites in the UK	Recruitment ongoing, started April 2022 (eConsent implemented prior to recruitment starting)	Trial comparing standard of care assessment pathway with pragmatic investigation based on contemporary risk stratification Primary outcome: composite of unobstructed coronary arteries on invasive angiography, myocardial infarction and cardiovascular death at a minimum of 12 months	Many cardiology clinics now conducted remotely since the COVID-19 pandemic	1055 participants recruited as of September 2023. 79 used eConsent (7%)
DOMINO-DFU	Diagnosis of osteomyelitis: investigation optimisation in diabetic foot ulcers—ISRCTN93847463	Cohort study incorporating three phases	Patients with a new Diabetic Foot Ulcer (DFU)	Diabetic foot ulcer clinics at three secondary care sites in the UK	Recruitment ongoing, started November 2021 (eConsent implemented after recruitment started)	Phase 1: observational Phase 2: comparison of 2 bone sampling techniques (diagnostic concordance) Phase 3: development of a diagnostic prediction model	Study also includes recruitment of a ‘full clinical cohort’, gathering routinely-collected data about all patients at each site (with their consent) in order to characterise the population at risk of diabetic foot osteomyelitis	593 participants recruited as of September 2023, 3 used eConsent (1%)
The LRRC-QoL study	Health-related Quality of Life and survivorship in Locally Recurrent Rectal Cancer—ISRCTN13692671	Mixed methods study	Patients with locally recurrent rectal cancer (LRRC)	Sites in 14 countries. eConsent or paper-based consent offered in UK, USA, Canada, Australia, New Zealand, Denmark and The Netherlands. Only paper option offered in India, Pakistan, France, Spain, Italy, Sweden and Singapore	Recruitment ongoing, started November 2020 (eConsent implemented after recruitment started)	Overall study aim: to validate an international patient-reported outcome measure to assess health-related quality of life	Different methods for consent and participation implemented, including eConsent, paper-based methods, and telephone. eConsent was utilised across all English-speaking sites, the Netherlands, and Denmark	213 participants recruited as of September 2023, 62 used eConsent (29%)

#### MODULATE

The MODULATE platform trial evaluating multiple drug and dietary treatments for diarrhoea in people with ulcerative colitis was due to open in March 2020 but was heavily delayed due to the COVID-19 pandemic. The pandemic had substantial impact upon UK clinical research delivery [13] but also provided opportunities for rethinking trial processes to better serve the NHS and target populations, as it shifted processes from in-person research delivery to remote. In addition, the group of patients that this study intended to treat, many of whom were taking immunosuppressant drugs, were instructed to shield and would have been unable to attend hospital appointments as part of the trial.

The MODULATE team responded to these challenges by implementing a remote participant pathway. A key part of this was the development of an eConsent process to enable participants to give consent remotely. Remote consent was further supported by other remote processes, including a self-referral pathway, trial visits conducted by online meeting or telephone, finger-prick blood and stool sample kits posted to participants’ homes, study IMP posted to participants from trial pharmacies and remote delivery of the dietary intervention.

As MODULATE was a CTIMP, there was additional regulatory guidance to adhere to in the process design [8]. The trial team were keen to maximise participant choice and offered a paper option for remote consent (supported by telephone calls). The eConsent process was articulated in an updated protocol and patient information sheet. The trial team worked with our patient and public involvement (PPI) contributor to help explain eConsent (and its implications for data storage) to trial participants.

MODULATE was the first CTRU CTIMP to employ remote eConsent and ensured that the process was approved by the Research Ethics Committee and MHRA, paving the way for future use in other CTIMPs at the CTRU. MODULATE was closed early in January 2023, due to delays resulting from COVID-19 to research set-up and delivery. The recruitment numbers are too low to draw generalisable conclusions about the eConsent system used in the trial.

#### CE-MARC 3

CE-MARC 3 recruits patients with suspected cardiac chest pain from NHS cardiology outpatient departments. CE-MARC 3 is a pragmatic trial in which patients are randomly allocated to different cardiac investigations, all of which are routine care within the NHS. Consequently, this is a low-risk trial for patients and the majority agree to participation. Using eConsent offers sites practicality and flexibility to work with the individual needs of patients. Since the COVID-19 pandemic, many outpatient departments run remote clinics, typically on the phone [14].

The use of eConsent allows patients from these remote clinics to be recruited and randomised quickly without additional visits or reliance on posted forms. It can also be used to allow a patient to complete consent after a face-to-face clinic if they need more time.

The numbers of participants who have consented using eConsent or paper in CE-MARC 3 are similar across some key demographic characteristics. Overall, 93% of participants have so far used paper to consent, and 7% eConsent (Table 1). These proportions are almost identical to the trial-wide totals when looking separately at male and female participants and when comparing the largest ethnic group (White) to other ethnic groups (Table 2). The median age of those using paper is 63, versus 62 for eConsent.

**Table 2:** eConsent usage by sex and ethnicity in the CE-MARC 3 clinical trial

**Characteristic**	***n***	**Participants consented using paper process, *n* (%)**	**Participants consented using eConsent, *n* (%)**
**Sex**			
Male	614	566 (92)	48 (8)
Female	441	410 (93)	31 (7)
**Ethnicity**			
White	843	784 (93)	59 (7)
Minority ethnic group	106	96 (91)	10 (9)
Ethnicity not known	106	96 (91)	10 (9)

Usage between sites is much more varied, from 0% to as many as 75% of consents being via eConsent at one site (see Table 3). This largely correlates with the extent to which sites recruit via remote clinics. Without an eConsent process, it would have been difficult for these sites to recruit, given the other options of postal, paper-based consent, or recruiting only via less frequent in-person clinics.

**Table 3:** Site-by-site summary of paper and eConsent use within the CE-MARC 3 clinical trial

	Participants consented using paper process, *n* (%)	Participants consented using eConsent, *n* (%)	**Total number of participants consenting**
Site 1	3 (25)	9 (75)	**12**
Site 2	70 (64)	40 (36)	**110**
Site 3	42 (81)	10 (19)	**52**
Site 4	79 (88)	11 (12)	**90**
Site 5	163 (97)	5 (3)	**168**
Site 6	274 (99)	4 (1)	**278**
Site 7	31 (100)	0 (0)	**31**
Site 8	18 (100)	0 (0)	**18**
Site 9	115 (100)	0 (0)	**115**
Site 10	69 (100)	0 (0)	**69**
Site 11	73 (100)	0 (0)	**73**
Site 12	39 (100)	0 (0)	**39**
**Total**	**976 (93)**	**79 (7)**	**1055**

For CE-MARC 3, eConsent has reduced site and CTRU administrative time as, unlike with paper consent forms, documents do not need to be scanned and securely sent to the CTRU for central monitoring purposes. There is less data cleaning required for eConsent as the system limits missing or erroneous data.

There have been occasional technical issues. A few participants have not received emails with links to the eConsent form (for various reasons), and some patients were unable to see all questions required to complete the form. Some participants omitted to click the box to confirm the PDF of the consent form was correct, so despite the full consent form being completed, REDCap showed the form as incomplete. Regular effort is required to check the status of incomplete forms and decide what action is required.

#### DOMINO-DFU

The DOMINO-DFU study seeks to recruit all new diabetic foot ulcer referrals to a cohort. The study team aim to recruit as ‘complete’ a cohort for each site as possible. Large numbers of patients may therefore be consented and recruited at each centre, potentially placing significant demands on recruiters. eConsent was implemented to facilitate maximum recruitment by offering a second consent pathway. This was hypothesised to suit recruiters, who would not be able to attend every clinic to complete face-to-face consent, and also patients, who can be registered retrospectively (i.e. following the clinic visit) without needing to return to the clinic to give consent.

To date, eConsent has not been used as much as anticipated in this study. Only two out of four sites are so far set up to use eConsent, and out of nearly 600 participants in the cohort, only three participants have consented using eConsent. Anecdotally, it appears that site preferences or needs, and unfamiliarity with the new system, may contribute to this lack of uptake. The patient population tends to be elderly and frail, with multiple comorbidities, and there is some evidence that, in some cases, older people are not offered eConsent due to assumptions that they might find it difficult to use [15]. Alternatively, sites may have been able to recruit more patients than predicted in clinics and therefore remote eConsent has not been needed as much as anticipated. Where eConsent has been used, positive feedback has been received from research staff that the process was easier than they had expected.

#### The LRRC-QoL study

The LRRC-QoL study is an international, mixed methods study consisting of three workstreams regarding health-related quality of life (HRQOL) and survivorship in locally recurrent rectal cancer. The study is recruiting in 14 countries (see Table 1). Initially, recruitment and consent for the study were undertaken using only paper-based methods. In the first 6 months of the study, a relatively low proportion of patients approached about the study consented to take part. PPI work and focus group meetings with participating teams both highlighted that eConsent could improve recruitment rates.

The REDCap eConsent system was first developed for English-speaking sites in the UK, USA, Canada, New Zealand, and Australia. Versions for sites in the Netherlands and Denmark were developed subsequently, with on-screen text translated into Dutch and Danish. However, there is currently no way to translate the built-in system text (e.g. the ‘submit’ button) into other languages. The clinicians supporting recruitment at the Dutch and Danish sites felt that, given relatively high levels of English ability in the populations of those countries, this would not be a significant barrier to recruitment. However, this conclusion was not reached in the other involved countries, so they used only paper-based consent processes (with the materials translated into the relevant local languages). We recognise that this issue would need to be addressed for translations to be implemented on larger international platforms.

The eConsent process developed for the LRRC-QoL study is different from the CTRU standard, due to the low-risk nature of this study. Rather than potential participants being sent a personalised eConsent link, after introducing the study to potential participants, sites give out an initial ‘open’ eConsent link. Patients fill in their details and record their consent to take part in the study. The central co-ordinating researcher at the CTRU then confirms eligibility and patients’ identity with each site and completes the enrolment process. This approach is suitable and manageable in this study but would not be appropriate or scalable to larger-scale, interventional research.

The LRRC-QoL study is ongoing at the time of writing and 29% of 213 recruits have consented and participated in the study via REDCap. More male participants (32%) have chosen to use eConsent than female (23%). The median age of those using eConsent is 62, versus 66 for paper. Further work is needed to understand if these observations reflect real differences in these groups, or just chance. However, offering a range of methods for participating, including online, via paper, or via telephone, has had a clear positive impact on study recruitment.

## Conclusions

From our experiences so far, we have noted some benefits of eConsent. eConsent suits some trial sites’ clinical practice, following adoption of more remote clinics following the COVID-19 pandemic. There is less scope for documentation error with eConsent than with paper-based processes, consequently reducing the amount of central monitoring checks required. Central monitoring is possible in an even more timely manner without the need for central collection of consent form copies. It can be used in international studies, although limitations on the ability to translate system wording can be a challenge.

## Suggestions for future research on eConsent

We have not yet reported data on costs of eConsent but would support further research in this area. A resource-use comparison with paper consent may not be straightforward. In our experience, set-up of remote eConsent methods has not been costly, but training and access management takes time, and maintaining the system involves systems developer resource. As consent processes at study level should not exclude participant groups, it seems inevitable that use of eConsent means maintaining at least two consent systems (i.e. eConsent and paper consent), which has resourcing implications too. There may be savings elsewhere, for example in the reduced central monitoring workload.

We have seen some variation in uptake of eConsent across studies, sites and participants and suggest that understanding this variation—including the relevance of patient and research staff factors—would be a useful topic for further study. Some of the same barriers may explain patient and site staff reluctance, i.e. unfamiliarity or lack of confidence in use of new technology. Alternatively, differences in how sites run patient services or in the integration with existing systems or technology may be a significant factor. Anecdotal feedback on our eConsent process from the sites that have used it has been positive, and the flexibility of having two consent methods may appeal as sites can use the method (or a combination of methods) that suits them and their patient populations best.

Although we cannot definitively say what effect the availability of eConsent has had on recruitment to CTRU trials, our offering eConsent in addition to paper-based consent methods might self-evidently imply that our consent processes are more likely to suit a wider range of individuals. This is particularly important in rare diseases, where numbers of eligible patients are limited. We have no direct feedback from participants about eConsent, though evidence available elsewhere suggests it may be acceptable, at least in principle, including in the specific context of the REDCap eConsent module [4, 10, 16–18]. We currently have no data to share on whether consent methods might impact trial retention, but we suggest this would also be a useful topic for future research.

Our case studies give a mixed picture about whether participants’ sex, ethnicity and age have had an impact on preferences for consent method. However, in the larger of the two studies with information to report, it appears the consent method has not differed substantially across some participant groups. These results are exploratory only, and should be interpreted with caution, pending more definitive research by us or others. eConsent may support inclusivity of certain groups, such as rural populations where patients may live long distances from their healthcare provider. Sites may in general be able to recruit from a larger geographical area. However, further research is needed to test these hypotheses.

## Limitations

We note some limitations in our current process, aside from those already mentioned above. We find that the system cannot be used as flexibly as paper-based systems where other signatories might be required, for example consent witnesses or principal investigator countersignature. The system does not easily allow for more than one consent form version to be implemented at the same time within a trial, for example if different sites are working to different versions during implementation of protocol amendments. Our experience is that consent form changes during a study are relatively rare.

Patient and public involvement is well established at the CTRU. Patients advised on patient-facing text within the system and on the suitability of using eConsent with different study populations. However, we might conceivably have done more to involve patient contributors in the overall process design.

Although all users receive system training and we have aimed to make the system user-friendly, occasionally users of all kinds may not use the system correctly, meaning the consent process cannot be completed via eConsent. Errors have, however, also been noted in the use of paper consent forms [19–22]. We have seen issues that have not occurred in paper-based processes, such as partial page completion meaning the eConsent process cannot be concluded. We do not have data available to compare error rates between paper-based consent and eConsent, but we suggest this would be a useful area of future research.

In the event of any issues with the system, it can be challenging for CTRU to provide technical support in real time (as CTRU staff are not directly involved in the recruitment process), meaning the paper backup is used more than it otherwise might. As REDCap is third-party software, limitations or issues are often not within CTRU control. The REDCap eConsent module is also only one part of the platform, so it is possible that a dedicated eConsent system may have more functionality, but this would undoubtedly come at additional cost.

## Future developments at CTRU

CTRU will make increasing use of eConsent, particularly for remotely delivered trials where it will be essential. It is conceivable that eConsent might become the default consent approach, but this is still a while away and we cannot yet envisage not having another option readily available (i.e. paper for those who want it). We are currently exploring how to adapt eConsent for use with in-person settings. We would still need to overcome the limitations set out above, but developments in technology or in the availability of suitable devices within the NHS might allow movement on this quicker than we might expect. We will also continue to react to any trends in clinical practice, for example growth or decline in use of remote clinics.

We have noted that site-level factors sometimes dictate uptake of eConsent and, given the need for trials to be patient-centred [23], we seek to remove this barrier by understanding challenges at sites and offering further support where we can. Use of methods such as those forming the QuinteT Recruitment Intervention [24], to understand barriers to recruitment and whether eConsent might help overcome these, may be beneficial.

As highlighted above, our standard eConsent setup does not currently provide information prior to consent or facilitate the consent discussion. We may eventually look to introduce the former more standardly, if the benefits of doing it this way make it worthwhile. However, given the easy availability of remote meeting software, we have no plans to incorporate a remote meeting into our eConsent solution. In conclusion, we successfully implemented a remote eConsent process at the CTRU in multiple studies with varied designs, populations and interventions. By sharing our approach and our experiences, we aim to help others less familiar with eConsent and contribute to the growing understanding of how eConsent methods can be put into practice.

## Data Availability

The data supporting this work are available on reasonable request. All requests will be reviewed by relevant stakeholders, based on the principles of a controlled access approach. Requests to access data should be made to CTRU-DataAccess@leeds.ac.uk in the first instance.

## Abbreviations

ALCOA+: A set of principles defining the characteristics of ‘source data’, namely that it be Attributable, Legible, Contemporaneous, Original, Accurate, Complete, Consistent, Enduring and Available
CTIMP: Clinical Trial of an Investigational Medicinal Product
CTRU: Clinical Trials Research Unit at the University of Leeds
eConsent: Electronic consent
HRA: Health Research Authority
HRQOL: Health-related quality of life
MHRA: Medicines and Healthcare products Regulatory Agency
NHS: National Health Service
PPI: Patient and public involvement
REDCap: Research Electronic Data Capture
